# Protein phosphatase NtPP2C2b and MAP kinase NtMPK4 act in concert to modulate nicotine biosynthesis

**DOI:** 10.1093/jxb/eraa568

**Published:** 2020-12-01

**Authors:** Xiaoyu Liu, Sanjay Kumar Singh, Barunava Patra, Yongliang Liu, Bingwu Wang, Jinsheng Wang, Sitakanta Pattanaik, Ling Yuan

**Affiliations:** 1 College of Agriculture, Shanxi Agricultural University, Taigu, Shanxi, China; 2 Department of Plant and Soil Sciences, and the Kentucky Tobacco Research and Development Center, University of Kentucky, University Drive, Lexington, KY USA; 3 Tobacco Breeding Center, Yunnan Academy of Tobacco Agricultural Sciences, Kunming, Yunnan, China; 4 Tsinghua University, China

**Keywords:** Alkaloid biosynthesis, gene regulation, hairy roots, MAP kinase, nicotine, protein phosphatase 2C, secondary metabolism, tobacco

## Abstract

Protein phosphatases (PPs) and protein kinases (PKs) regulate numerous developmental, defense, and phytohormone signaling processes in plants. However, the underlying regulatory mechanism governing biosynthesis of specialized metabolites, such as alkaloids, by the combined effects of PPs and PKs, is insufficiently understood. Here, we report the characterization of a group B protein phosphatase type 2C, NtPP2C2b, that likely acts upstream of the *NICOTINE2* locus APETALA 2/Ethylene Response Factors (AP2/ERFs), to regulate nicotine biosynthesis in tobacco. Similar to the nicotine pathway genes, *NtPP2C2b* is highly expressed in roots and induced by jasmonic acid (JA). Overexpression of *NtPP2C2b* in transgenic hairy roots or stable transgenic tobacco plants repressed nicotine pathway gene expression and reduced nicotine accumulation. Additionally, transient overexpression of *NtPP2C2b*, together with the *NtERF221*, repressed transactivation of the *quinolinate phosphoribosyltransferase* promoter in tobacco cells. We further demonstrate that the JA-responsive tobacco mitogen-activated protein kinase (MAPK) 4 interacts with NtPP2C2b in yeast and plant cells. Conditional overexpression of *NtMPK4* in tobacco hairy roots up-regulated nicotine pathway gene expression and increased nicotine accumulation. Our findings suggest that a previously uncharacterized PP-PK module acts to modulate alkaloid biosynthesis, highlighting the importance of post-translational control in the biosynthesis of specialized plant metabolites.

## Teaser

A previously uncharacterized protein phosphatase (NtPP2C2b)-MAP kinase (NtMPK4) module regulates nicotine biosynthesis, highlighting the importance of post-translational control in plant alkaloid biosynthesis.

## Introduction

**Fig. 1.**
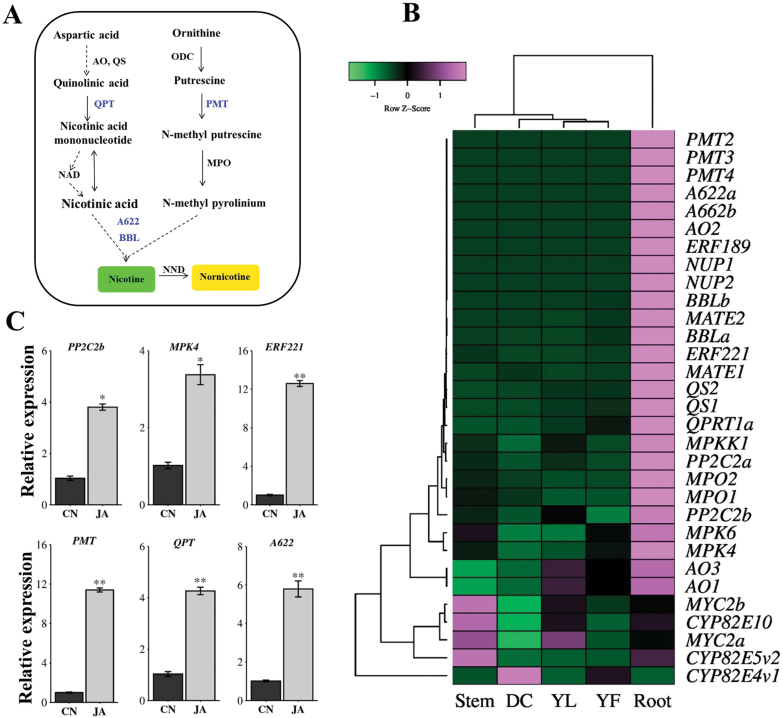
Co-expression analysis and JA-induced expression of *NtPP2C2b*, *NtMPK4* and nicotine pathway genes. (A) Schematic diagram of nicotine biosynthetic pathway in tobacco. A622, isoflavone reductase-like protein; ADC, arginine decarboxylase; AO, aspartate oxidase; BBL, berberine bridge enzyme-like; ODC, ornithine decarboxylase; MPO, N-methylputrescine oxidase; NND, nicotine N-demethylase QPT, quinolinate phosphoribosyltransferase; QS, quinolinate synthase; PMT, putrescine N-methyltransferase. (B) Co-expression analysis of *NtPP2C*, *NtMPK4* and nicotine pathway genes was performed using leaf, root, stem and flower transcriptomes (accession no. PRJNA208209). Correlation heatmap showing *NtPP2C2* and *NtMPK4* co-expressing with nicotine pathway genes. (C) Relative expression of *NtPP2C2b*, *NtMPK4* and other nicotine pathway genes in control (CN) and JA-treated roots. Relative expression was measured using real-time quantitative PCR (RT–qPCR). Tobacco *EF1œ* was used as an internal control. Data represent mean ±SD of three biological samples. Statistical significance was calculated using the Student’s *t*-test: *, *P*<0.05; **, *P*<0.01.

*Nicotiana tabacum* (common tobacco) is an allotetraploid that likely originated from *N. sylvestris* and *N. tomentosiformis* 200 000 years ago (Wang and Bennetzen, 2015). Nicotine is a major alkaloid accounting for more than 90% of the total alkaloid content in tobacco, which is synthesized in roots and transported to leaves (Dewey and Xie, 2013). Nicotine comprises a pyridine ring and a pyrrolidine ring which are derived from aspartate and ornithine, respectively (Fig. 1A; Dewey and Xie, 2013). Genes encoding the key enzymes in the nicotine biosynthetic pathway have been characterized (Dewey and Xie, 2013; Wang and Bennetzen, 2015). In addition, transcription factors (TFs) belonging to two major families, the APETALA2/ETHYLENE RESPONSE FACTORS (AP2/ERFs) and the basic helix-loop-helix (bHLH) factor MYC2, have been identified as positive regulators of nicotine biosynthesis (Shoji *et al.*, 2010; De Boer *et al.*, 2011; Shoji and Hashimoto, 2011; Zhang *et al.*, 2012; Sui *et al.*, 2019; Hayashi *et al.*, 2020). Two *AP2/ERF* gene clusters, presumably originated from its two diploid ancestors, have been identified. The *NICOTINE2* (*NIC2)* cluster, which originates from *N. tomentosiformis*, comprises at least 12 AP2/ERFs, whereas another cluster originating from *N. sylvestris* harbors six ERFs (Kajikawa *et al.*, 2017). The *NIC2* genes *NtERF189* and *NtERF221* (also known as ORC1), as well as the homologous *NtERF199* encode major transcription factors that regulate nicotine biosynthesis (Kajikawa *et al.*, 2017; Hayashi *et al.*, 2020). NtERF189 binds to the GC-rich motifs in the promoters of nicotine biosynthetic pathway genes to regulate their expression (Shoji *et al.*, 2010). Overexpression of *NtERF189* and *NtERF221* in tobacco increases nicotine accumulation, whereas CRISPR-Cas9-mediated knockout of *NtERF189/199* reduces accumulation of nicotine (De Boer *et al.*, 2011; Hayashi *et al.*, 2020). NtERF221 activates the promoters of multiple nicotine pathway genes, including *putrescine N-methyltransferase* (*PMT*) and *quinolinate phosphoribosyltransferase* (*QPT*), mediated by both GC-rich and G-box motifs in the promoters (De Boer *et al.*, 2011; Paul *et al.*, 2020). The bHLH TF NtMYC2 regulates expression of the *NIC2* locus AP2/ERFs and co-regulates nicotine biosynthetic pathway genes concurrently with the ERFs (Shoji and Hashimoto, 2011). The JASMONATE ZIM-DOMAIN (JAZ) proteins, known to bind and sequester MYC2, are negative regulators of nicotine biosynthesis (Shoji *et al.*, 2008). Similar to many other plant specialized metabolites, nicotine biosynthesis is induced by jasmonic acid (JA) and its methyl ester, methyl jasmonate (MeJA). The JA-induced expression of nicotine biosynthetic pathway genes and nicotine accumulation is inhibited by RNAi-mediated silencing of the JA receptor, CORONATINE INSENSITIVE1 (COI1), further substantiating the role of JA in transcriptional regulation of the nicotine biosynthetic pathway (Shoji *et al.*, 2008).

In addition to transcriptional control, metabolic pathways are regulated at the post-transcriptional and translational levels (De Boer *et al.*, 2011; Li *et al.*, 2015; Patra *et al.*, 2013a; Paul *et al.*, 2017; M. Zhang *et al.*, 2018; Zhang *et al.*, 2019). Although our understanding on transcriptional regulation of nicotine biosynthesis has significantly advanced over the years, post-translational regulation of the pathway is insufficiently understood. Phosphorylation by protein kinases (PKs) and dephosphorylation by protein phosphatases (PPs) are the major post-translational regulatory mechanisms that control biological processes in plants. Amongst PKs, the mitogen-activated protein kinases (MAPKs) are well characterized and known to be involved in growth, development, phytohormone signaling, and biotic and abiotic stresses (Hamel *et al.*, 2006). The MAPK cascade comprises of at least three functionally related kinases, mitogen-activated protein kinase kinase kinase (MAPKKK), mitogen-activated protein kinase kinase (MAPKK), and MAPK, which are involved in signal amplification, integration and transmission in response to specific stimuli (Hamel *et al.*, 2006). MAPK cascade components have been identified in many plant species; however, very few of them have been functionally characterized for their roles in specialized metabolism. A previous study has shown that transient overexpression of a MAPKK from tobacco, *JAM1* (*JA-FACTOR STIMULATING MPKK1*), together with *NtERF221* and/or *bHLH1,* in tobacco cells significantly up-regulates the promoter activity of key nicotine pathway genes, *PMT* and *QPT* (De Boer *et al.*, 2011). However, the effect of *JAM1* overexpression on nicotine accumulation is not known. Recently, we have shown that overexpression of a MAPKK, *CrMPKK1*, in *Catharanthus roseus* hairy roots up-regulates the expression of terpenoid indole alkaloid (TIA) pathway genes and boosts TIA accumulation (Paul *et al.*, 2017). In addition, transient overexpression of the *C. roseus* MAPK3 (*CrMPK3*) in leaves was also shown to induce TIA pathway gene expression and increase TIA accumulation (Raina *et al.*, 2012). In Arabidopsis, AtMPK3 and AtMPK6 phosphorylate the WRKY TF, WRKY33 to regulate the biosynthesis of camalexin, a phytoalexin that plays a major role in disease resistance (Mao *et al.*, 2011). A recent study has demonstrated that differential phosphorylation of WRKY33 by CALCIUM-DEPENDENT PROTEIN KINASE5 (CPK5)/CPK6 and MPK3/MPK6 regulates camalexin biosynthesis in Arabidopsis (Zhou *et al.*, 2020). 

The kinase activity of MAPKs is regulated by a large family of PPs in plants (Schweighofer *et al.*, 2004). PPs are divided into two major groups: serine/threonine phosphatases and protein tyrosine phosphatases (PTPs). The serine/threonine phosphatases are further divided into phosphoprotein phosphatases (PPP), and metal-dependent protein phosphatases (PPM) that include protein phosphatase type 2C (PP2C; Schweighofer *et al.*, 2004; Singh *et al.*, 2016). The group B PP2Cs are key regulators of MAPK activity in plants such as Arabidopsis and alfalfa (*Medicago sativa*) (Schweighofer *et al.*, 2004). The alfalfa MP2C (Medicago protein phosphatase 2C) is the first PP2C identified as a regulator of MAPK activity (Meskiene *et al.*, 1998; Meskiene *et al.*, 2003). The Arabidopsis genome harbors 76 PP2Cs which are sub-divided into ten groups (A-J). The group B PP2C, AP2C1 interacts with, and inactivates AtMPK4 and AtMPK6 to regulate JA and ethylene biosynthesis, thus modulating innate immunity (Schweighofer *et al.*, 2007). However, the combined influence of PP2C and MAPK on alkaloid pathway gene expression and accumulation has not been explored.

Here, we report the analysis of the tobacco PP2C family, functional characterization of NtPP2C2b, and demonstration of the interaction between NtPP2C2b and NtMPK4 in regulation of nicotine biosynthesis. Similar to nicotine pathway genes, *NtPP2C2b* is highly expressed in roots and induced in response to JA treatment. Overexpression of *NtPP2C2b* in transgenic hairy roots or tobacco plants altered nicotine pathway gene expression and nicotine accumulation. In addition, NtPP2C2b interacts with the JA-responsive MAP kinases, NtMPK4 and NtMPK6, and the two genes encoding these kinases also co-expressed with the nicotine pathway genes. Overexpression of *NtMPK4,* but not *NtMPK6,* altered nicotine pathway gene expression in tobacco hairy roots. The complementary effects of a JA-responsive MAPK and a protein phosphatase on nicotine biosynthesis highlight the existence of a post-translational regulatory mechanism that likely acts upstream of AP2/ERFs to fine-tune nicotine biosynthesis in tobacco.

## Materials and methods

### Plant material and treatments

*Nicotiana tabacum* ‘Samsun NN’ was used for gene cloning and generation of hairy roots and transgenic plants. *N. tabacum* ‘Xanthi’ cell line was used for protoplast-based transient expression assays. For phytohormone induction, tobacco hairy roots were separately treated with 100 μM methyl jasmonate (MeJA), 100 μM abscisic acid (ABA), 100 μM ethylene (ET) precursor ACC (1-aminocyclopropane-1-carboxylic acid), 50 μM auxin (2,4-dichlorophenoxyacetic acid; 2,4-D), or 20 μM salicylic acid (SA) for 2 h. For DEX (dexamethasone, Sigma-Aldrich USA) treatment of pTA7001-*NtMPK4* lines, 30 μM DEX was added to Murashige and Skoog (MS) medium, and the hairy roots were incubated for 12 h before being used for RNA isolation and metabolite measurement (Paul *et al.,* 2020).

### Vector construction, generation of transgenic hairy roots and plant transformation

For generation of transgenic hairy roots and tobacco plants, *NtPP2C2b, NtMPK4,* and *NtMPK6* were PCR amplified from tobacco root cDNA, and cloned into pCAMBIA2301 vector containing a *CaMV*35S promoter and the *rbcS* terminator (Paul *et al*., 2020). The pCAMBIA2301 vector alone was used as an empty vector (EV) control. For conditional overexpression, *NtMPK4* was cloned in a pTA7001 vector under the control of a DEX-inducible promoter (Aoyama and Chua, 1997). For generation of hairy roots, the plasmids were mobilized into *Agrobacterium rhizogenes* R1000 by freeze-thawing. Transformation of tobacco leaf discs and generation of hairy roots were performed using the protocol described previously (Paul *et al.*, 2020). Transgenic status of the hairy root lines was verified by PCR amplification of *rolB*, *rolC*, *virC*, and *nptII* genes. For generation of stable transgenic plants, the plasmids were mobilized into *Agrobacterium tumefaciens* GV3850. Tobacco leaf disc transformation and generation of transgenic plants was performed as previously described (Pattanaik *et al.*, 2008). Primers used in this study are listed in Supplementary Table S1. Two independent hairy roots or transgenic tobacco lines were selected for further analysis.

### Reverse transcription–quantitative PCR

Total RNA isolated from tobacco seedlings, leaves or hairy roots using RNeasy Plant Mini Kit (QIAGEN, USA) was used for cDNA synthesis and RT–qPCR, as previously described (Paul *et al.*, 2017). The 2^-∆∆CT^(cycle threshold) method (Livak and Schmittgen, 2001) was used to quantify gene expression. In addition to the tobacco *elongation factor1α* (*EF1α*; GenBank accession number D63396; Shoji *et al.*, 2010), *α-tubulin* (GenBank accession number AJ421411; Paul *et al.*, 2020) was also used as an internal control. The primers used in RT–qPCR are listed in Supplementary Table S1.

### Vector construction, protoplast isolation, and electroporation

The reporter plasmids for transient protoplast assays were generated by cloning the *NtPMT* and *NtQPT* promoters upstream of a firefly *luciferase* (*LUC*) and *rbcS* terminator (Paul *et al.*, 2020). The effector plasmids were made by cloning *NtERF221, NtPP2C2a, NtPP2C2b*, and *NtMPK4* into a modified pBS vector under the control of the *CaMV*35S promoter and *rbcS* terminator. The ß-glucuronidase (*GUS*) gene driven by the *CaMV*35S promoter and *rbcS* terminator was used as an internal control in the protoplast assay. For plant cell-based interaction assays (Patra *et al.*, 2013a), NtMPK4 was fused to the GAL4 DNA binding domain (GAL-BD) in a pBS plasmid containing the *mirabilis mosaic virus* (*MMV*) promoter and *rbcS* terminator. The reporter plasmid used in the assay contains firefly *LUC* driven by minimal *CaMV* 35S promoter with five tandem repeats of *GAL4 Response Elements* (*5×GALRE*), and *rbcS* terminator. Protoplast isolation from tobacco cell suspension cultures and electroporation with plasmid DNA were performed as described previously (Pattanaik *et al.*, 2010). The reporter, effector, and internal control plasmids were electroporated into tobacco protoplasts in different combinations; luciferase and GUS activities in transfected protoplasts were measured as described previously (Pattanaik *et al.*, 2010). Each experiment was repeated three times.

### Vector construction and yeast two-hybrid assay

The full-length cDNA of *NtMPK4* or *NtMPK6* was cloned into pAD-GAL4-2.1, and *NtPP2C2a* or *NtPP2C2b* was cloned into pBD-GAL4 Cam (Stratagene, USA). The plasmids were transformed into yeast strain AH109 using the PEG/LiCl method (Clontech, USA), and transformed cells were selected on synthetic dropout (SD) medium lacking leucine and tryptophan (-leu-trp). Transformed colonies were then streaked on SD medium lacking histidine, leucine, tryptophan (-his-leu-trp) to verify protein-protein interactions.

### Identification of *PP2C* and *MAPK* genes in tobacco and phylogenetic analysis

To identify the *PP2C* genes in *N. tabacum*, amino acid sequences of all Arabidopsis PP2Cs were retrieved from the TAIR database (https://www.arabidopsis.org), based on a previous report (Xue *et al.*, 2008). These sequences were then used as queries to perform Basic Local Alignment Search Tool (BLAST; Camacho *et al.*, 2009) searches with default settings against the *N. tabacum* reference sequences downloaded from the Sol Genomics Network database (Fernandez-Pozo *et al.*, 2015). Subsequently, the putative tobacco PP2Cs were identified using the PP2C model (Pfam accession number PF00481; http://pfam.xfam.org/) and HMMER software (http://hmmer.org/), and the proteins without a PP2C catalytic domain were deleted. The full-length protein sequences of phosphatase genes were aligned using ClustalW with default settings (Larkin *et al.*, 2007). MEGA6.0 (Tamura *et al.*, 2013) was used to construct the phylogenetic tree using the neighbor-joining (NJ) method with bootstrap values set as 1000 replicates.

For identification of the MAPK gene family, the sequences of Arabidopsis MAPK cascade proteins were obtained from TAİR (https://www.arabidopsis.org/browse/genefamily/MAPK.jsp). These sequences were used as queries to search against the tobacco protein sequences by the BLASTP program with the default parameters. The tobacco proteome was also searched for the MAPK-specific motif T[E/D]YVxTRWYRAPE[L/V], as described previously (Jonak *et al.*, 2002). The unique sequences obtained from the above-mentioned methods were further verified using HMMSCAN (https://www.ebi.ac.uk/Tools/hmmer/search/hmmscan) to confirm the presence of a MAPK domain (PF00069). Furthermore, the putative tobacco MAPK sequences were reciprocally searched against the Arabidopsis database to identify the best hit amongst all the MAPK genes. Finally, the sequences that did not contain the known conserved domains were removed.

### Transcriptome analysis

To determine the spatiotemporal expression of *NtPP2C*, *NtMPK4* and *NtMPK6* genes in *N. tabacum*, RNA-seq data of different tissues (leaf, stem, root, flower and capsule) were obtained from the sequence read archive database (SRA, accession number PRJNA208209; Sierro *et al.*, 2014). Raw Illumina sequence reads were processed using the prinseq-lite-0.20.4 (Schmieder and Edwards, 2011) to remove low-quality reads (Singh *et al.*, 2015). Subsequently, pre-processed reads were assessed for quality control using FastQC (version 0.11.3; Babraham Bioinformatics, Cambridge, UK). Read mapping was performed by Bowtie2 (Langmead and Salzberg, 2012) using the reference sequence downloaded from the Sol Genomics Network database (Fernandez-Pozo *et al.*, 2015). The log_2_ Fragments Per Kilobase of transcript per Million mapped reads (FPKM) was used for clustering calculation and visualization by ‘stats’ and ‘gplots’ packages in R (https://www.R-project.org/; https://CRAN.R-project.org/package=gplots).

### Agrobacterium infiltration of *N. benthamiana* leaves, immunoblotting and dephosphorylation assay

For transient expression, *NtPP2C2b* fused to *enhanced GFP* (*eGFP*) was cloned in pCAMBIA2301 containing the *CaMV* 35S promoter and *rbcS* terminator. *NtMPK4* fused to a 3×FLAG epitope was cloned in pCAMBIA1300 with the *CaMV*35S promoter and *rbcS* terminator. *Agrobacterium tumefaciens* GV3101 cells harboring pCAMBIA1300-NtMPK4-FLAG were infiltrated alone or in combination with pCAMBIA2301-NtPP2C-eFGP into four week-old *Nicotiana benthamiana* leaves. Leaf samples were collected after 48 h of infiltration, and total proteins were extracted using extraction buffer containing 10% glycerol, 25 mM Tris-Hcl, pH 7.5, 150 mM NaCl, and 1× protease inhibitor cocktail (ThermoFisher Scientific, USA). Western blotting was performed using anti-FLAG-M2 (Sigma, USA; Cat # F3165) and anti-GFP (ThermoFisher Scientific, USA; Cat # MA1-052) antibodies to detect the NtMPK4-FLAG and NtPP2C-eGFP proteins, respectively. Next, we used the anti-FLAG-M2 Affinity gel (Sigma; Cat # A2220) to immunoprecipitate the NtMPK4-FLAG protein following the manufacturer’s protocol. The SuperSep PHOS-tag gel (Wako, Japan; Cat # 19517991; Nagy *et al.*, 2018) was used to separate the immunoprecipitated NtMPK4-FLAG protein, followed by western blotting using Anti-FLAG-M2 (Sigma; Cat # F3165) antibody, according to the manufacturer’s protocol.

### Alkaloid extraction and analysis

Nicotine content in hairy roots and tobacco leaves overexpressing *NtPP2C2b, NtMPK4* and empty vector was measured using Gas Chromatography with Flame Ionization Detectors (GC-FID; PerkinElmer, USA; Paul *et al.*, 2020). Nicotine content was reported as mg g^-1^ on a dry weight basis.

## Results and discussion

### Genome-wide identification of *NtPP2C* genes in tobacco

*PP2C* is an evolutionarily conserved gene family found in archaea, bacteria, and plants. *Marchantia polymorpha* (liverwort) ABSCISIC ACID INSENSITIVE1 (MpABI1) is probably the most ancient PP2C characterized for its role as a negative regulator of ABA signaling (Hauser *et al.*, 2011). To identify putative *PP2C* genes in *N. tabacum*, the Pfam PP2C domain ‘PF00481’ was used to search the tobacco proteome. We also performed a BLAST search of the tobacco proteome using reported Arabidopsis PP2C proteins as queries. After the removal of redundant sequences, protein hits with the conserved PP2C catalytic domains were considered as putative PP2C family members. A total of 164 full-length proteins were identified as putative PP2C family members, compared with 130 PP2Cs in maize (Wei and Pan, 2014). The genome of a lower plant moss (*Physcomitrella*) contains 51 *PP2C* genes, but the number increases significantly in higher plants, with Arabidopsis and rice having 76 and 90 *PP2Cs*, respectively (Schweighofer *et al.*, 2004; Singh *et al.*, 2010; Hauser *et al.*, 2011). Plant genomes harbor more *PP2C* genes than yeast and mammals; for example, the human genome contains 16 *PP2C* genes that encode for 22 isozymes (Lammers and Lavi, 2007). These findings suggest that the increase and diversification of *PP2Cs* have occurred during the course of plant evolution (Singh *et al.*, 2016). 

The 164 putative *NtPP2C* genes identified in this study encode proteins varying from 137 to 1083 amino acids in length, with isoelectric point (pI) values ranging from 4.13 to 9.73, and molecular masses ranging from 15.07 kDa to 121.22 kDa (Supplementary Table S2). Phylogenetic analysis showed that NtPP2C proteins can be divided into 16 sub-groups (A-P; Supplementary Fig. S1). We were particularly interested in Group B PP2Cs because of their ability to regulate MAPKs and their involvement in phytohormone signaling. The Arabidopsis Group B PP2Cs, AP2C1, AP2C2 and AP2C3 (Schweighofer *et al.*, 2007; Umbrasaite *et al.*, 2010) and alfalfa MP2C (Meskiene *et al.*, 1998, 2003) are known regulators of MAPK activity in plants. In tobacco, Group B contains 10 members which are clustered together with six Group B members of Arabidopsis in a phylogenetic tree (Supplementary Fig. S1). We identified two tobacco PP2Cs which are homologous to the well-characterized group B PP2Cs in alfalfa (Meskiene *et al.*, 1998, 2003) and Arabidopsis (Schweighofer *et al.*, 2004). They share 49–52% amino acid sequence identity with alfalfa (MP2C) and Arabidopsis (AP2C1/2/3), and are designated here as NtPP2C2a and NtPP2C2b (Supplementary Fig. S2). NtPP2C2a and NtPP2C2b are 90% identical in their amino acid sequences and presumably originated from *N. tometosiformis* and *N. sylvestris*, respectively, the two progenitors of tobacco. The group B phosphatases from tobacco, Arabidopsis, and alfalfa contain the kinase interaction motif (KIM; K/R_[3–4]_-X_[1–6]_-L/I) at the amino terminus (Supplementary Fig. S2), which is important for interaction with MAPKs (Schweighofer *et al.*, 2007). In addition, sequence alignment also revealed that amino acid residues important for phosphate and metal ion binding are well conserved in PP2Cs of tobacco, Arabidopsis, and alfalfa (Supplementary Fig. S2; Das *et al.*, 1996). We speculated that NtPP2C2a and NtPP2C2b interact with MAPKs in JA signaling, which governs nicotine biosynthesis.

### *NtPP2C2* co-expresses with nicotine pathway genes and is inducible by jasmonic acid

Nicotine is synthesized in roots and translocated to leaves through the vasculature. The expression of functionally related genes often show similar spatial and temporal expression patterns in many specialized metabolic pathways (Mutwil, 2020). The genes encoding nicotine biosynthetic pathway enzymes, such as *NtPMT* and *NtQPRT1* (Fig. 1A), and the *NIC2* locus ERFs are preferentially expressed in roots (Kajikawa *et al.*, 2017). We thus reasoned that genes associated with the regulation of nicotine biosynthesis exhibit similar expression patterns. To determine the spatial expression of *NtPP2C2* (*NtPP2C2a* and *NtPP2C2b*), we used the available transcriptomic resources (Accession no. PRJNA208209) to perform co-expression analysis. As shown in the heatmap (Fig. 1B), similar to the nicotine pathway genes, both *NtPP2Cs* are preferentially expressed in roots. As both *NtPP2Cs* share high sequence identity and similar expression profile, *NtPP2C2b* was selected for further characterization.

JA is a major elicitor of biosynthesis of many specialized metabolites in plants, such as steroidal glycoalkaloids (SGA) in tomato (Nakayasu *et al.*, 2018), terpenoid indole alkaloids in *C. roseus* (Paul *et al.*, 2020), and nicotine in tobacco (Shoji *et al.*, 2008, 2010). An increase in amounts of endogenous JA or exogenous application of JA induces the expression of major regulatory and enzyme-encoding genes in the nicotine pathway. To determine whether *NtPP2C2* is induced by JA, we performed RT–qPCR on RNA isolated from control (untreated) and JA-treated tobacco roots. The expression of *NtPP2C2b* was induced four-fold in response to JA treatment, compared with controls. As expected, expression of *NtERF221*, *NtPMT*, and *NtQPT* was induced four to twelve-fold relative to controls, following JA treatment (Fig. 1C). The results signify the involvement of *NtPP2C2b* in JA signaling. In addition, we measured the expression of *NtPP2C2b* using RT–qPCR in tobacco roots separately treated with SA, ABA, ACC (a precursor of ethylene), or 2,4-D (a synthetic auxin); two internal reference genes, *EF1α* and *α-tubulin,* were used to normalize gene expression. *NtPP2C2b* showed slight induction in expression (approximately two-fold) with all phytohormones (Supplementary Fig. S3A, B), suggesting possible roles in other biological processes. Removal of terminal buds (also known as topping) is a common agronomic practice that significantly impacts the yield and quality of various crop plants including tobacco. The stable expression of reference genes was validated by measuring the expression of three different transcription factor genes in control and topped tobacco leaves (Supplementary Figs S3C, D).

### *NtPP2C2b* overexpression alters the expression of genes regulating nicotine accumulation in hairy roots

**Fig. 2.**
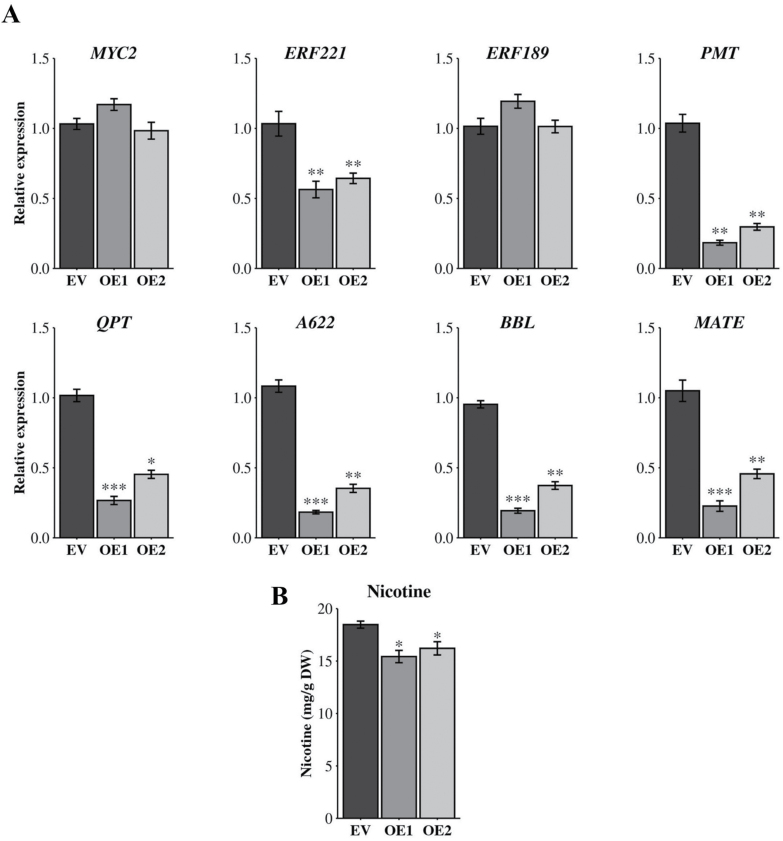
Expression analysis of nicotine pathway genes and nicotine accumulation in *NtPP2C2b*-overexpressing hairy roots. (A) Relative expression of regulatory (*MYC2, ERF221*and *ERF189* ) and enzyme-encoding (*PMT, QPT, A622, BBL* and *MATE*) genes in the nicotine pathway in empty vector (EV) and *NtPP2C2b*-overexpressing hairy roots (OE1 and OE2). Relative expression was measured using RT–qPCR. Tobacco *EF1*α was used as an internal control. (B) Nicotine content in EV and *NtPP2C2b*-overexpressing hairy roots (OE1 and OE2) was measured using gas chromatography-flame ionization detector (GC-FID) and presented as mg g^-1^ dry weight (DW). Data represent mean ±SD of three biological samples. Statistical significance was calculated using the Student’s *t*-test: *, *P*<0.05; **, *P*<0.01. ***, *P*<0.001.

To determine the regulatory roles of *NtPP2C2b* on nicotine pathway gene expression and nicotine accumulation, we generated transgenic tobacco hairy roots overexpressing (OE) *NtPP2C2b*. The transgenic status of the hairy roots was verified by RT–PCR (Supplementary Fig. S4A). Two *NtPP2C2b*-overexpressing transgenic lines (OE1 and OE2) and one empty vector (EV) line were selected for further analysis. The expression of *NtPP2C2b* was 12–18 fold higher in OE lines compared with the EV line (Supplementary Fig. S4B). We measured the expression of key regulators, *NtMYC2a*, *NtERF221*, and *NtERF189*, as well as genes encoding nicotine pathway enzymes, *NtPMT*, *NtQPT*, *NtBBL* (*berberine bridge enzyme-like*), and *NtA622,* in EV and OE lines. PMT catalyzes the conversion of putrescine to N-methylputrescine in the first committed step of nicotine biosynthesis. N-methylputrescine serves as precursor for the pyrrolidine moiety of nicotine. QPT is the major enzyme for synthesis of the pyridine moiety. BBL and A622 catalyse the coupling of pyridine and pyrrolidine rings in later steps of nicotine biosynthesis (Fig. 1A; Yuan, 2020). In the OE lines, expression of *NtERF221* was reduced by 40% whereas expression of *NtMYC2a* and *NtERF189* did not change significantly (Fig. 2A). In addition, expression of key nicotine pathway genes, such as *NtPMT*, *NtQPT*, *NtA622*, and *NtBBL,* was reduced by 52–76% in *NtPP2C2-*OE lines (Fig. 2A). Nicotine is sequestered into the root vacuoles by the MATE (multidrug and toxic compound extrusion) transporters in tobacco (Shoji *et al.*, 2009). Expression of *NtMATE* was also reduced by 53–74% in both OE lines relative to EV. To determine the metabolic outcome resulting from *NtPP2C2b* overexpression, we measured nicotine content in two OE and EV tobacco hairy root lines. Nicotine accumulation was moderately reduced (about 15%) in both transgenic lines relative to EV control (Fig. 2B).

### *NtPP2C2b* overexpression represses the expression of genes regulating nicotine accumulation in transgenic tobacco

**Fig. 3.**
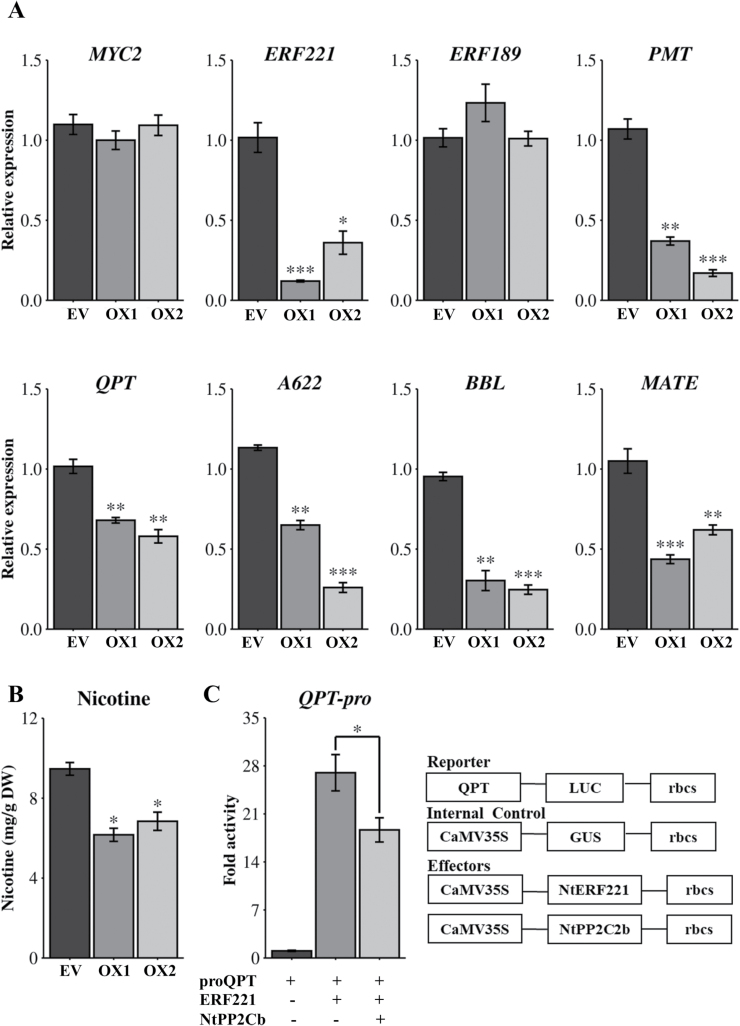
Expression analysis of nicotine pathway genes and nicotine accumulation in *NtPP2C2b*-overexpressing transgenic tobacco plants, and promoter assay in tobacco cells. (A) Relative expression of regulatory (*MYC2, ERF221*and *ERF189* ) and enzyme-encoding (*PMT, QPT, A622, BBL* and *MATE*) genes in the nicotine pathway in empty vector (EV) and transgenic tobacco plants overexpressing *NtPP2C2b* (OX1 and OX2). Relative expression was measured using RT–qPCR. Tobacco *EF1*α was used as an internal control. (B) Nicotine content in leaves of EV and transgenic tobacco plants overexpressing *NtPP2C2b* (OX1 and OX2) measured using GC-FID and presented as mg g^-1^ dry weight (DW). (C) Co-expression of *NtPP2C2b* with *NtERF221* repressed the *NtQPT* promoter (*proQPT*) activity in tobacco cells. pro*QPT* fused to the firefly *luciferase* (*LUC*) reporter was co-electroporated into tobacco cells alone or with effector plasmids expressing *NtERF221* and *NtPP2C2b* in different combinations. A plasmid containing *CaMV*35S-GUS was used as an internal control. LUC activity was normalized against the GUS activity. Schematic diagrams of reporter, effector and internal control plasmids are shown. Data represent mean ±SD of three biological samples. Statistical significance was calculated using the Student’s *t*-test: *, *P*<0.05; **, *P*<0.01. ***, *P*<0.001.

To further substantiate the role of *NtPP2C2b* in regulation of the nicotine pathway, we generated stable transgenic tobacco plants overexpressing *NtPP2C2b* or the EV. Transgenic status of the tobacco lines was verified by RT–PCR (Supplementary Fig. S4C). Two independent *NtPP2C2b* overexpressing lines (OX1 and OX2), showing 18–25 fold higher expression than EV, were used for further analysis (Supplementary Fig. S4D). Similar to transgenic hairy roots, expression of *ERF221,* and nicotine pathway genes, including *NtPMT*, *NtQPT*, *NtBBL,* and *NtMATE,* was reduced by 38–82% in two OX lines compared with EV (Fig. 3A). In addition, nicotine content in two overexpression lines was reduced by 28–35% compared with the EV line (Fig. 3B).

The reduced expression of nicotine pathway genes suggest that *NtPP2C2b* overexpression in hairy roots and transgenic tobacco plants likely alters the phosphorylation magnitude of key regulators, such as ERF221, and affects their transcriptional activity. Notably, expression of *NtERF221,* but not *NtERF189,* was reduced in the transgenic roots (Fig. 3A). A previous study has shown that although *NtERF221* is a close functional homolog of *NtERF189* and both are involved in nicotine biosynthesis, basal expression of *NtERF221* is significantly lower compared with *NtERF189* (Kajikawa *et al.*, 2017). In addition, while *NtERF189* expression was gradually induced by JA and repressed by NaCl, expression of *NtERF221* is rapidly and significantly induced both by JA and NaCl (Kajikawa *et al.*, 2017). The differences in basal expression and response to JA and other factors indicate that the two genes are differentially regulated. As expression of *NtMYC2* was not changed in *NtPP2C2b*-OE lines, we speculate that the activities of regulators controlling *MYC2* expression are not regulated by NtPP2C2b. Although the expression of key nicotine pathway genes reduced significantly, we observed a moderate reduction of nicotine content in both hairy roots and transgenic plants. It is not surprising, because the tobacco *nic1, nic2, and nic1nic2* mutants, in which the key regulatory genes, such as *NtERF221*, *NtERF189*, *NtERF199*, are deleted, still accumulate low amounts of nicotine (Hibi *et al.*, 1994). Furthermore, it is possible that additional candidate PPs are potentially involved in the regulatory network.

### Transient overexpression of *NtPP2C2b* represses *quinolinate phosphoribosyltransferase (QPT)* promoter activity in tobacco cells

To determine the effects of NtPP2C2b on transactivation of nicotine pathway gene promoters by NtERF221, we performed protoplast-based transient transactivation assay. Tobacco *QPT* promoter fused to firefly *luciferase* reporter was electroporated into tobacco protoplasts with vectors expressing *NtERF221* and *NtPP2C2b*, alone or in combination. As expected, NtERF221 alone significantly induced *NtQPT* promoter activity (28-fold) compared with controls. However, co-expression of *NtERF221* with *NtPP2C2b* reduced the promoter activity compared with expression of *NtERF221* alone, suggesting that NtPP2C2b negatively affects the activity of NtERF221 in tobacco cells (Fig. 3C). As promoter activity was assayed in tobacco cells, we hypothesized that NtERF221 is likely phosphorylated by an endogenous MAPK, such as NtMPK4 which is altered by co-expression of *NtPP2C2b*, resulting in reduced activity of the target *QPT* promoter.

### Genome-wide identification of MAPKs in tobacco

The group B PP2C are known to physically interact with MAPKs to regulate the kinase activity (Meskiene *et al.*, 1998, 2003; Schweighofer *et al.*, 2007; Umbrasaite *et al.*, 2010). We hypothesized that NtPP2C2b interacts with tobacco MAPKs. A previous study has identified 17 MAPKs in tobacco based on expressed sequence tags (ESTs; Zhang *et al.*, 2013). The recent availability of the tobacco genome compelled us to examine further the tobacco *MAPK* (*NtMPK*) gene family. Our genome-wide analysis resulted in the identification of 29 *NtMPK* genes, of which 15 genes were identified previously (Zhang *et al.*, 2013). NtMPKs were classified into four classes (A-D) as described previously (Jonak *et al.*, 2002). MAPKs are activated through phosphorylation of the highly conserved threonine and tyrosine residues (-TxY-motif; x presenting random amino acid) in the activation loop (T-loop; Camps *et al.*, 2000). Similar to Arabidopsis MAPKs, tobacco MAPKs in groups A, B, and C possess a TEY-motif in the T-loop, while group D MAPKs contain a TDY-motif (Supplementary Fig. S5; Ichimura *et al.*, 2002). Other variants of the TxY-motif, such as T(Q/V/S)Y, T(/Q/R)M, MEY, TEC and TEM, have also been reported in the T-loop (Mohanta *et al.*, 2015). We found that, in addition to the TEY/TDY motifs, NtMPK2 and NtMPK27 contain the MEY motif in the T-loop. The details of *NtMPK* genes are given in Supplementary Table S3.

In tobacco, NtMPK3, NtMPK4, and NtMPK6 have been characterized for their roles in JA signaling, as well as in wounding and defense responses (Liu *et al.*, 2003; Gomi *et al.*, 2005). The group B PP2C AP2C1 in Arabidopsis interacts with and dephosphorylates AtMPK4 and AtMPK6 to regulate JA and ethylene biosynthesis, to modulate innate immunity (Schweighofer *et al.*, 2007). Similar to AtMPK4, NtMPK4 belongs to group B whereas NtMPK6 grouped together with AtMPK6 in group A. In addition, NtMPK4 and NtMPK6 share more than 85% amino acid sequence identity with AtMPK4 and AtMPK6, respectively (Supplementary Fig. S6). We thus hypothesized that NtMPK4 and NtMPK6 are targets of NtPP2C2b and involved in nicotine biosynthesis. We next determined the spatial and JA-induced expression of *NtMPK4* and *NtMPK6* and interaction of these protein kinases with NtPP2C2b in yeast and plant cells.

### *NtMPK4* co-expresses with nicotine pathway genes and is inducible by jasmonic acid

*NtMPK4* is highly expressed in roots (Fig. 1B). To determine whether expression of *NtMPK4* is inducible by JA, we measured *NtMPK4* expression using RT–qPCR in control and JA-treated hairy roots. Similar to *NtPP2C2b* and nicotine pathway genes, expression of *NtMPK4* was induced about three-fold in response to JA treatment (Fig. 1C). Similar spatial expression of *NtMPK4* with *NtPP2C2b* and nicotine pathway genes is indicative of its involvement in nicotine biosynthesis.

### NtPP2C2b interacts with NtMPK4

**Fig. 4.**
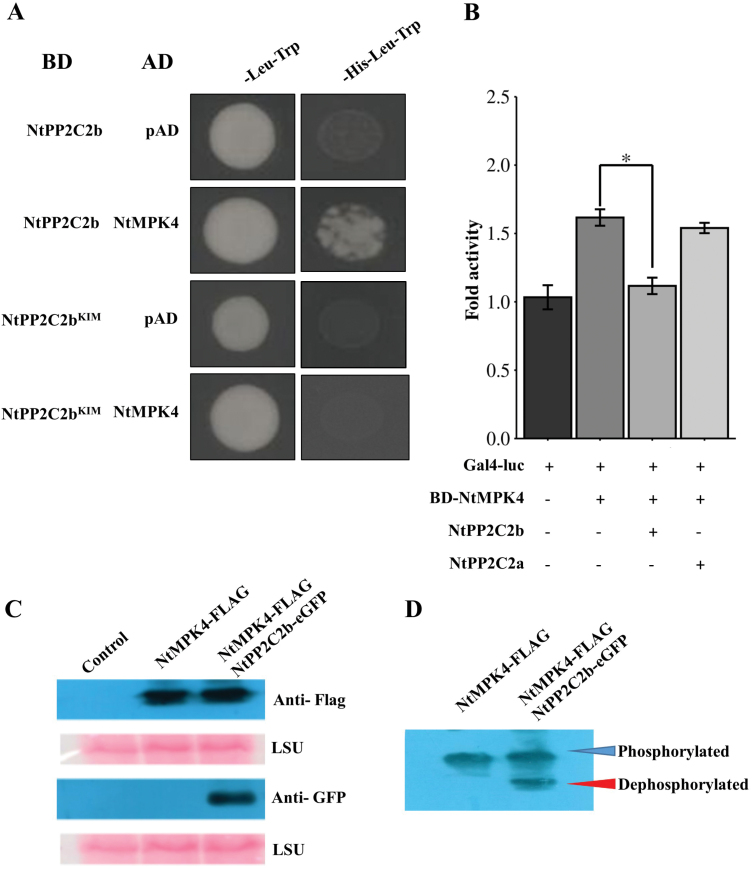
NtPP2C2b interacts with NtMPK4 in yeast and plant cells. (A) NtPP2C2b interacts with NtMPK4 in yeast cells. Mutation in NtPP2Cb KIM (K89A R90Q; mNtPP2Cb) abolished interaction with NtMPK4. NtPP2C2b fused to GAL4 DNA binding domain and NtMPK4 fused to GAL4 activation domain were transformed into yeast strain AH109. Protein-protein interaction was detected by the growth of the cells on triple (-his-leu-trp) selection medium. (B) Protoplast-based assay shows that NtPP2C2b interacts with NtMPK4 in tobacco cells. NtMPK4 fused to the GAL4 DNA binding domain (BD-NtMPK4) was electroporated into tobacco protoplasts alone or in combination with NtPP2C2a or NtPP2C2b, and luciferase reporter. The *CaMV*35-GUS plasmid was used as an internal control. Luciferase activity was normalized against GUS activity. Each experiment was repeated three times. Statistical significance was calculated using the Student’s *t*-test: *, *P*<0.05. (C) *NtMPK4-FLAG* alone or in combination with *NtPP2C2b-eGFP* was transiently expressed in *N. benthamiana* leaves. The proteins were detected using anti-GFP or anti-FLAG antibodies in western blot. (D) The transiently produced proteins were immunoprecipitated, separated on a PHOS-tag gel, and detected using anti-FLAG antibodies. The phosphorylated (higher band) and dephosphorylated (lower band) NtMPK4 showed differential migration on the PHOS-tag gel. The Rubisco large sub-unit (LSU), stained by PonceauS, is shown as a loading control.

We next performed yeast two-hybrid assays to determine whether NtPP2C2a and NtPP2C2b interact with NtMPK4. As shown in Fig. 4A, NtPP2C2b interacts with NtMPK4 in yeast. However, NtPP2C2a does not interact with NtMPK4 (Supplementary Fig. S3E). We next used a protoplast-based assay to confirm the interaction (Mitsuda and Ohme-Takagi, 2009; Patra *et al.*, 2013a). NtMPK4, fused to the GAL4 DNA binding domain (BD) alone or in combination with NtPP2C2a or NtPP2C2b, was electroporated into tobacco protoplasts. As shown in Fig. 4B, BD-NtMPK4 activated the *luciferase* reporter driven by minimal *CaMV*35S promoter with GAL-response elements. Luciferase activity was reduced significantly when *NtPP2C2b* was co-expressed with BD-NtMPK4, indicating an interaction between the two proteins expressed in plant cells; however, *NtPP2C2a* had no effect on NtMPK4 activation of the luciferase reporter. The interaction between Arabidopsis AP2C1 and AtMPK4 depends on an intact KIM, and mutation in the KIM weakens or abolished the interaction with PP (Schweighofer *et al.*, 2007). Amino acid sequence analysis revealed the presence of a putative KIM in NtPP2Cb (Supplementary Fig. S2). Mutation of the conserved lysine to alanine (K89A), and arginine to glutamine (R90Q) in the KIM sequence of NtPP2C2b abolished its interaction with NtMPK4 (Fig. 4A). KIM is also present in MAPKKs in plants. A MAPKK interacts with and phosphorylates the MAPKs, which is mediated by KIM (Kiegerl *et al.*, 2000; Paul *et al.*, 2017). The presence of a similar motif (i.e. KIM) in MAPKKs and group B PP2Cs indicate that the two enzymes probably compete to bind to the substrate MAPK in response to specific stimuli, and balance the activation of downstream targets.

To determine whether the NtPP2C2b-NtMPK4 interaction leads to dephosphorylation of NtMPK4, we transiently expressed *NtMPK4-FLAG* alone or in combination with *NtPP2C2b-eGFP* in *N. benthamiana* leaves, followed by detection using anti-GFP or anti-FLAG antibody in a western blot (Fig. 4C). Once the target proteins were detected by western blot analysis, the NtMPK4 protein was immmunoprecipitated from leaves expressing *NtMPK4-FLAG* alone or *NtMPK4-FLAG* with *NtPP2C2b-eGFP,* and detected using an anti-FLAG antibody in a western blot. As shown in Fig. 4D, immunoprecipitated proteins from *NtMPK4*-infiltrated leaves showed a single band, whereas proteins from co-infiltrated leaves showed two distinct bands, one of which displayed decreased molecular mass. Therefore, NtMPK4 is likely to be autophosphorylated in plant cells, and co-expression of *NtPP2C2b* with *NtMPK4* dephosphorylated the NtMPK4 protein. The dephosphorylated NtMPK4 therefore migrated faster in the PHOS-tag gel (Fig. 4D).

### Conditional overexpression of *NtMPK4* in hairy roots induces the expression of genes regulating nicotine accumulation

**Fig. 5.**
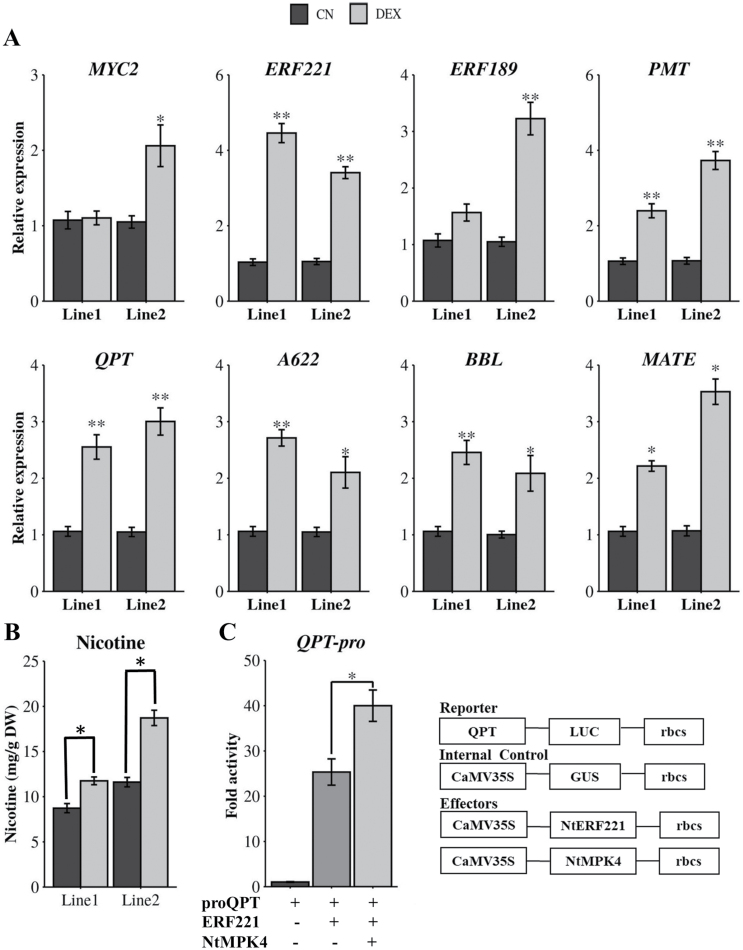
Expression analysis of nicotine pathway genes and nicotine accumulation in *NtMPK4*-overexpressing hairy roots and promoter assay in tobacco cells. (A) Relative expression of regulatory and enzyme-encoding genes in the nicotine pathway in control (CN) and dexmethoasone-treated (DEX) *NtMPK4*-overexpressing hairy roots (LINE1 and LINE2). Relative expression was measured using RT–qPCR. Tobacco *EF1*α was used as an internal control. (B) Nicotine content in control (CN) and dexmethasone-treated (DEX) *NtMPK4*-overexpressing hairy roots (LINE1 and LINE2) measured using GC-FID and presented as mg g^-1^ dry weight (DW) basis. (C) Co-expression of NtPP2C2b with NtMPK4 increased the *NtQPT* promoter (*proQPT*) activity in tobacco cells. pro*QPT* fused to the firefly *luciferase* (*LUC*) reporter was co-electroporated into tobacco cells alone or with the effector plasmids expressing *NtERF221*, and *NtMPK4* in different combinations. A plasmid containing *CaMV*35S-GUS was used as an internal control. LUC activity was normalized against the GUS activity. Data represent mean ±SD of three biological samples. Statistical significance was calculated using the Student’s *t*-test: *, *P*<0.05; **, *P*<0.01.

To determine the function of NtMPK4 in nicotine biosynthetic pathway gene expression and nicotine accumulation, we generated transgenic tobacco hairy roots overexpressing *NtMPK4*. We have observed that constitutive expression of *NtMPK4* driven by the *CaMV*35S promoter resulted in a delay in hairy root initiation and stunted root growth. To alleviate this problem, we overexpressed *NtMPK4* using a DEX-inducible promoter (Aoyama and Chua, 1997). We generated more than 10 transgenic lines in which *NtMPK4* expression was induced using DEX. Transgenic status of the roots was verified by PCR (Supplementary Fig. S4E); two *NtMPK4* overexpression lines (Line 1 and Line 2) showing two to three-fold induction (Supplementary Fig. S4F) compared with the non-induced lines were selected for further analysis. Next, we measured the expression of key regulators, nicotine pathway genes and a transporter of nicotine biosynthesis in transgenic lines. Expression of *NtERF221*, *NtPMT*, *NtQPT*, and *NtBBL* was induced two to four-fold in DEX-induced *NtMPK4*-overexpression lines compared with the non-induced lines (Fig. 5A). Furthermore, expression of *NtMATE* was also induced in DEX-induced lines relative to controls. We measured the nicotine content in DEX-induced and non-induced lines. As shown in Fig. 5B, nicotine accumulation was increased by 35–62% in DEX-induced lines compared to the non-induced control. Nicotine content in the two non-induced control (CN) lines were different as they represent two independent transgenic lines.

### Transient overexpression of *NtMPK4* activates the *QPT* promoter in tobacco cells

We performed protoplast transient transactivation assay to determine the effects of NtMPK4 on NtERF221 activity. Tobacco *QPT* promoter fused to firefly *luciferase* reporter was electroporated into tobacco protoplasts along with vectors expressing *NtERF221* or *NtMPK4* as effectors, alone or in combination (Fig. 5C). As expected, NtERF221 induced *NtQPT* promoter activity by 26-fold. Co-expression of *NtERF221* with *NtMPK4* resulted in a further increase in *NtQPT* promoter activity (35-fold), suggesting that NtMPK4 potentially increased the activity of NtERF221 by phosphorylation (Fig. 5C).

It is well known that phosphorylation affects the stability, DNA binding properties, and activity of regulatory proteins (Menke *et al.*, 2005; Zhai *et al.*, 2013; Li *et al.*, 2017; Zhai *et al.*, 2017). Here, increased activation of the *NtQPT* promoter in tobacco cells by combined expression of *NtMPK4* and *NtERF221*, and elevated expression of nicotine pathway genes in DEX-induced *NtMPK4*-overexpression lines suggest that NtERF221 is likely to be phosphorylated by NtMPK4, leading to an increase in transcriptional activity of NtERF221 and/or binding to its target promoters (Fig. 5A, C). Expression of *NtERF221* also increased in NtMPK4-overexpressing hairy root lines (Fig. 5A), suggesting that NtMPK4 probably phosphorylates a regulator(s), other than NtMYC2, that activates *ERF221* expression. Increased expression of nicotine pathway genes leads to higher nicotine accumulation in roots (Fig. 5B). We propose that NtMPK4 forms a module with NtPP2Cb to modulate nicotine biosynthesis. A PK-PP module regulates biosynthesis of artemisinin, a sesquiterpene lactone, in *Artemisia annua* through the bZIP factor, AabZIP1 (M. Zhang *et al*., 2018; Zhang *et al*., 2019). The abscisic acid (ABA)-responsive sucrose non-fermenting 1-related kinase (SnRK) AaAPK1 phosphorylates AabZIP1 and enhances its transactivation activity on artemisinin pathway genes (F. Zhang *et al.*, 2018). AaAPK1 itself is dephosphorylated by interacting with the ABA-responsive group A PP2C, AaPP2C1 (Zhang *et al.*, 2019). In addition, AaPP2C1 negatively affects the transactivation of AabZIP1 on promoters of artemisinin pathway genes. These findings suggest that similar to the artemisinin pathway, nicotine biosynthesis is regulated by concerted actions of PP-PK and downstream regulatory proteins.

### Jasmonic acid-inducible NtMPK6 interacts with NtPP2C2b but does not influence the nicotine pathway

Similar to *NtMPK4* and *NtPP2C2b*, expression of *NtMPK6* was also induced by JA (Supplementary Fig. S7A). In addition, NtMPK6 interacted with NtPP2C2b in yeast cells (Supplementary Fig. S7B). We next generated transgenic hairy roots overexpressing *NtMPK6* (Supplementary Fig. S7C) and measured the expression of two key nicotine pathway genes, *NtPMT* and *NtQPT* in EV and two overexpression lines. Expression of both genes remained unchanged in the two transgenic lines (Supplementary Fig. S7D). These findings suggest that the NtPP2C2b-NtMPK6 module is not involved in nicotine pathway regulation, but rather, plays other roles in the JA signaling pathway.

Conclusion

In conclusion, many specialized metabolites, such as alkaloids, are produced in response to various biotic and abiotic stimuli. As the biosynthesis of specialized metabolites is an energy-demanding process, plants have evolved multiple layers of regulatory mechanisms to fine-tune their accumulation. This is achieved at the transcriptional level by concerted actions of transcriptional activators and repressors through (i) protein-protein interactions and/or (ii) competitive binding to target gene promoters, subsequently affecting gene expression and metabolic outcomes. This regulatory mechanism is evident in flavonoid and terpenoid indole alkaloid pathways in plants (Matsui *et al.*, 2008; Patra *et al.*, 2018; Sui *et al.*, 2018). Many of these activators and repressors are also regulated at post-translational levels by PPs and PKs. The magnitude and duration of MAPK activity are crucial for proper activation of developmental, defense or other metabolic pathways. MAPK signaling needs to be deactivated to prevent overstimulation of a pathway, and to maintain homeostasis (Jiang *et al.*, 2017). 

**Fig. 6.**
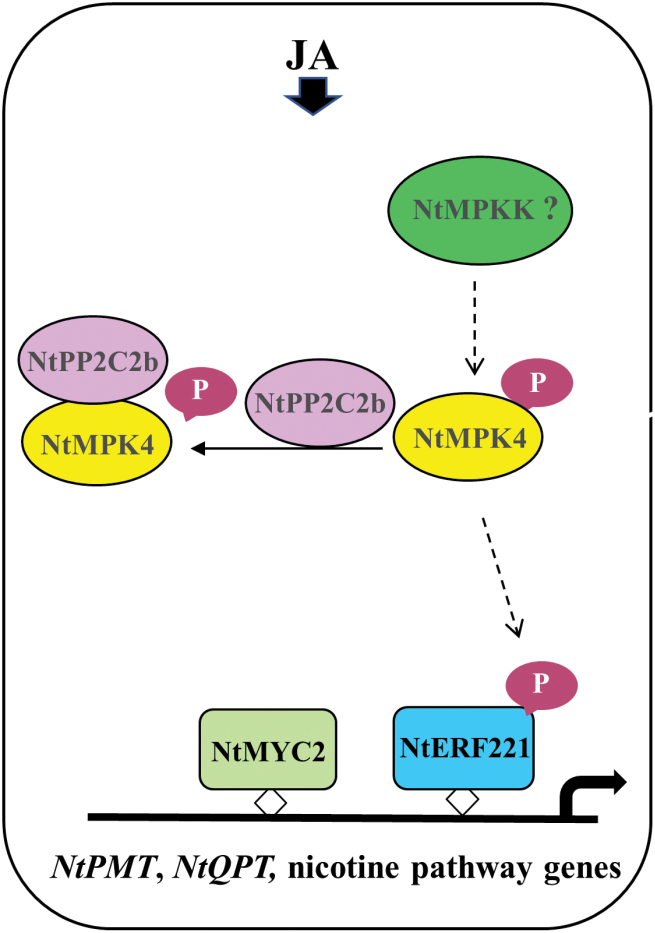
A simple model depicting the regulation of the nicotine biosynthetic pathway by NtPP2C2b and NtMPK4 in tobacco. Expression of both *NtMPK4* and *NtPP2C2b* is induced by JA. NtMPK4 is phosphorylated and activated by an unidentified MAPKK (NtMPKK, indicated by dotted arrow). Activated NtMPK4 likely phosphorylates the *NIC2* locus ERFs (NtERF221 and possibly other ERFs in the locus), which, along with NtMYC2 regulates the expression of nicotine pathway genes (such as *NtPMT*, *NtQPT*). NtPP2C2b interacts with, and likely dephosphorylates NtMPK4 to maintain the homeostasis between phosphorylated and dephosphorylated forms of NtMPK4, thereby fine-tuning the activation of genes regulating nicotine accumulation.

We demonstrated that NtMPK4 positively, whereas NtPP2C2b negatively regulates nicotine biosynthesis (Figs 2, 3, 5). JA-induced expression of phosphatase and kinase is required to fine-tune the accumulation of nicotine. Although many PKs and PPs have been characterized for their roles in regulating developmental, stress signaling, and defense-related pathways (Schweighofer *et al.*, 2004; M. Zhang *et al.*, 2018), regulation of specialized metabolite biosynthesis by PPs and PKs has not been well studied. Prior to this study, co-regulation of alkaloid biosynthesis by a PK-PP module has not been reported. Here, we performed genome-wide analysis to identify all putative PP2Cs and MAPKs in tobacco, which provides a valuable resource for functional characterization of candidate PPs and PKs for their roles in specialized metabolism or other biological processes. Additionally, we demonstrated that similar to the nicotine pathway genes, *NtPP2C2b* and *NtMPK4* are highly expressed in roots and induced by JA (Fig. 1). Overexpression of *NtMPK4* activates, whereas that of *NtPP2C2b* represses, nicotine biosynthesis in tobacco (Figs 2, 3, 5). Moreover, NtPP2C2b interacts with NtMPK4 in yeast and plant cells (Fig. 4). We suggest that NtMPK4 acts upstream of ERF221 to increase its activity on target promoters such as *QPT*. The group B PP2Cs are known to interact and dephosphorylate MAPKs to regulate kinase activities (Meskiene *et al.*, 2003; Schweighofer *et al.*, 2007). We demonstrated that NtPP2C2b dephosphorylates NtMPK4 (Fig. 4), which in turn affects the activity of ERF221 (Fig. 6). The complementary effects of kinase and phosphatase suggest that a phosphorylation-dephosphorylation mechanism regulates nicotine biosynthesis. NtPP2C2b and NtMPK4 act in concert to regulate specific genes in the nicotine pathway, e.g. *ERF221,* but not *ERF189* or *NtMYC2*. A limited number of PKs and PPs have been characterized for their roles in regulating plant metabolic pathways, and very few direct substrates for NtMPK4 or NtPP2Cs have been identified. An in-depth analysis of the tobacco phosphoproteome using emerging techniques will potentially lead to the identification of direct substrates for candidate PKs and PPs. In addition, future biochemical evidence on reversible protein phosphorylation will shed more light on its regulation of the alkaloid biosynthetic pathway. Accumulating evidence suggests that the regulatory mechanisms and the regulators of many structurally similar or diverse specialized metabolites are evolutionarily conserved across species. For example, biosynthesis of flavonoids is regulated by a conserved group of R2R3MYB, bHLH, and WD40 proteins in a wide range of plant species, including Arabidopsis and tobacco (Patra *et al.*, 2013b). A small group of JA-responsive AP2/ERFs regulates structurally diverse terpenoid indole alkaloids in *Catharanthus*, nicotine in tobacco, and steroidal glycoalkaloids in tomato and potato (Shoji *et al.*, 2010; Cardenas *et al.*, 2016; Paul *et al.*, 2017; Nakayasu *et al.*, 2018; Paul *et al.*, 2020). Therefore, our findings lay the foundation for exploring PP-PK regulatory modules in other specialized metabolic pathways. Identification of PPs and PKs will further advance our understanding of the regulation of specialized metabolic pathways in plants.

## Supplementary data

The following supplementary data are available at *JXB* online.

Fig. S1. Phylogenetic analysis of tobacco and Arabidopsis PP2Cs.

Fig. S2. Amino acid sequence alignment of tobacco, Arabidopsis and alfalfa PP2Cs.

Fig. S3. Relative expression of *NtPP2C2b* in roots treated with ABA, ACC, auxin and SA.

Fig. S4. Molecular analysis of hairy roots and transgenic tobacco plants.

Fig. S5. Phylogenetic analysis of MAPKs from tobacco and Arabidopsis.

Fig. S6. Amino acid sequence alignment of Arabidopsis and tobacco MPK4 and MPK6.

Fig. S7. Expression, protein-protein interaction, and molecular analysis of *NtMPK6*-overexpressing hairy roots.

Table S1. Primers used in this study.

Table S2. Details of tobacco *PP2C* genes

Table S3. Details of tobacco MAP kinase genes

eraa568_suppl_Supplementary_Material

eraa568_suppl_Supplementary_Table_S2_S3

## Data Availability

All data supporting the findings of this study are available within the paper and within its supplementary data published online.
