# Supplementary material for: Protein phosphatase NtPP2C2b and MAP kinase NtMPK4 act in concert to modulate nicotine biosynthesis
Source: J Exp Bot. 2020 Dec 1;72(5):1661–76. doi: 10.1093/jxb/eraa568 (PMC7921305; doi:10.1093/jxb/eraa568)
Supplement: eraa568_suppl_Supplementary_Material [file eraa568_suppl_supplementary_material.pdf]

Supplementary information:

Protein-phosphatase NtPP2C2b and MAP-kinase NtMPK4 act in concert to modulate nicotine biosynthesis

Xiaoyu Liu[1,2], Sanjay Kumar Singh[2], Yongliang Liu[2], Bingwu Wang[3], Jinsheng Wang[1]

Sitakanta Pattanaik[2] and Ling Yuan[2]

[1]College of Agriculture, Shanxi Agricultural University, Taigu, 030801, Shanxi, China

[2]Department of Plant and Soil Sciences and the Kentucky Tobacco Research and Development Center, University of Kentucky, 1401 University Drive, Lexington, KY 40546 USA

[3]Tobacco Breeding Center, Yunnan Academy of Tobacco Agricultural Sciences, Kunming 650021, Yunnan, China

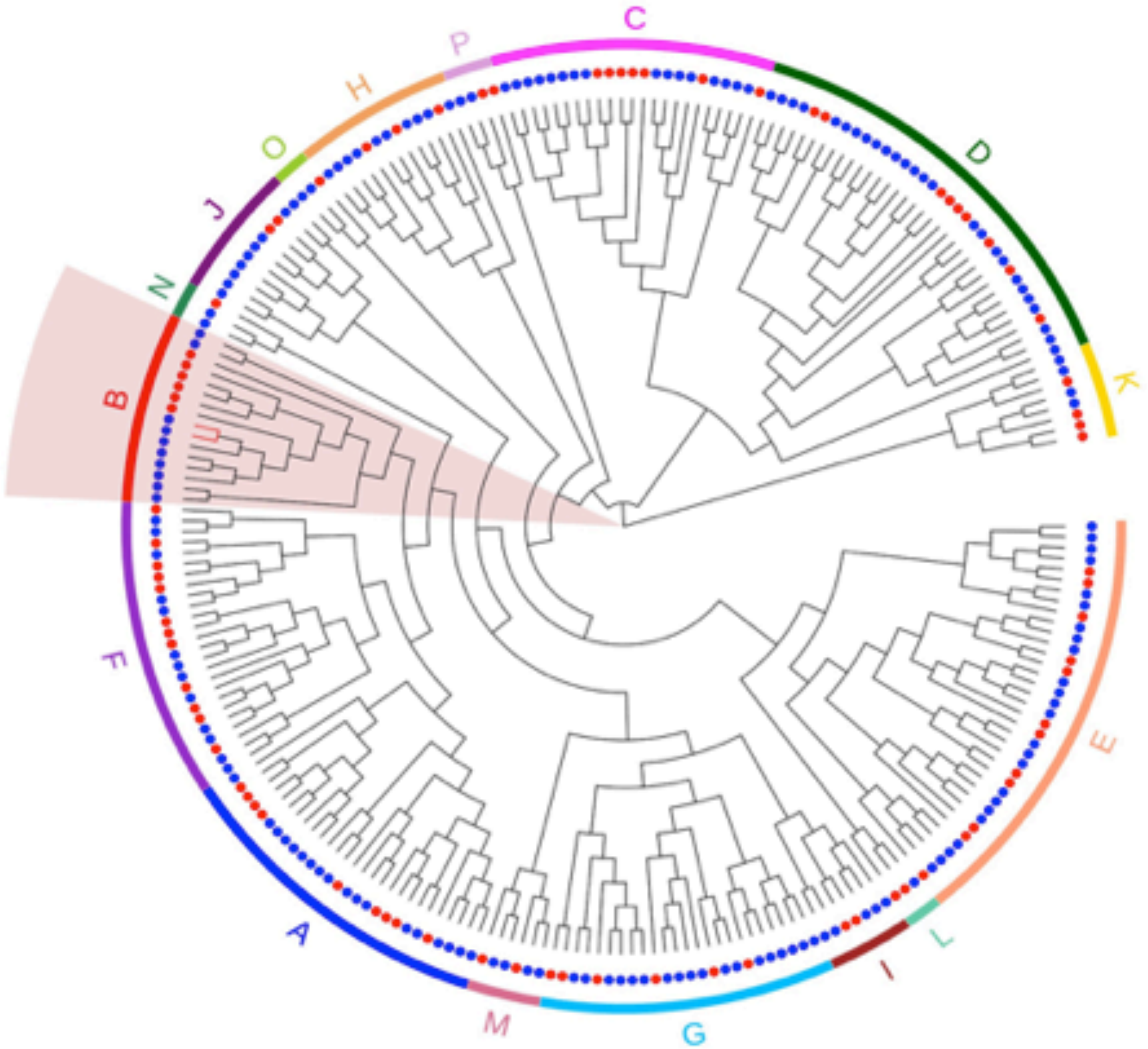

Fig. S1 Phylogenetic analysis of tobacco and Arabidopsis protein phosphatase type 2C (PP2C). The PP2Cs from *Arabidopsis thaliana* and *Nicotiana tabacum* (tobacco) were used for analysis. MEGA6.0 was used to construct the phylogenetic tree with the bootstrap values set as 1000 replicates. The evolutionary history was inferred using the neighbor-joining method. Tobacco PP2Cs are indicated by *blue* while *Arabidopsis* ones are by *red* dots. Group B clade is highlighted with light-red color.

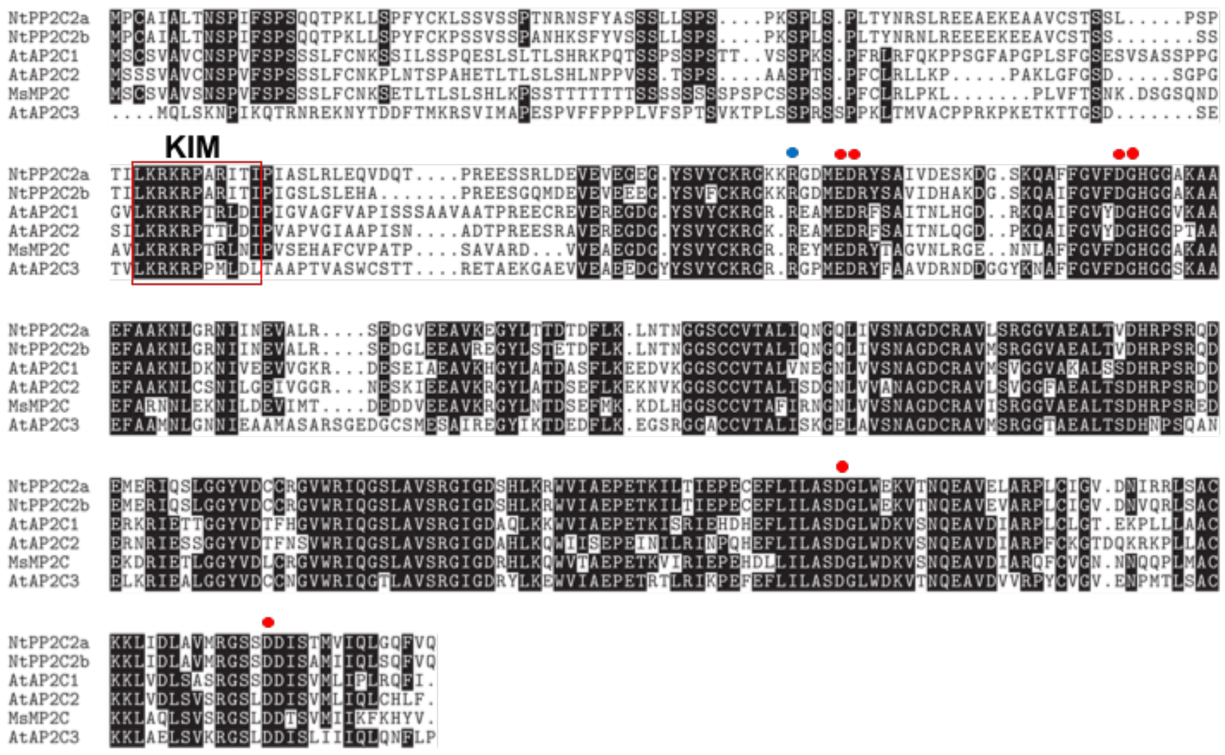

Fig. S2 Amino acid sequence alignment of tobacco, Arabidopsis and alfalfa PP2Cs. Alignment of tobacco (NtPP2C2a and NtPP2C2b), Arabidopsis (AtAP2C1, AtAP2C2 and AtAP2C3) and alfalfa (*Medicago sativa*; MsMP2C) PP2Cs was performed with the CLUSTALO program using the default settings. Identical amino acid residues in the alignment are highlighted in black. The location of the conserved kinase interaction motif (KIM) is indicated by rectangle (red). Residues putatively involved in the phosphate and metal ion binding are indicated by *blue* and *red* dots, respectively.

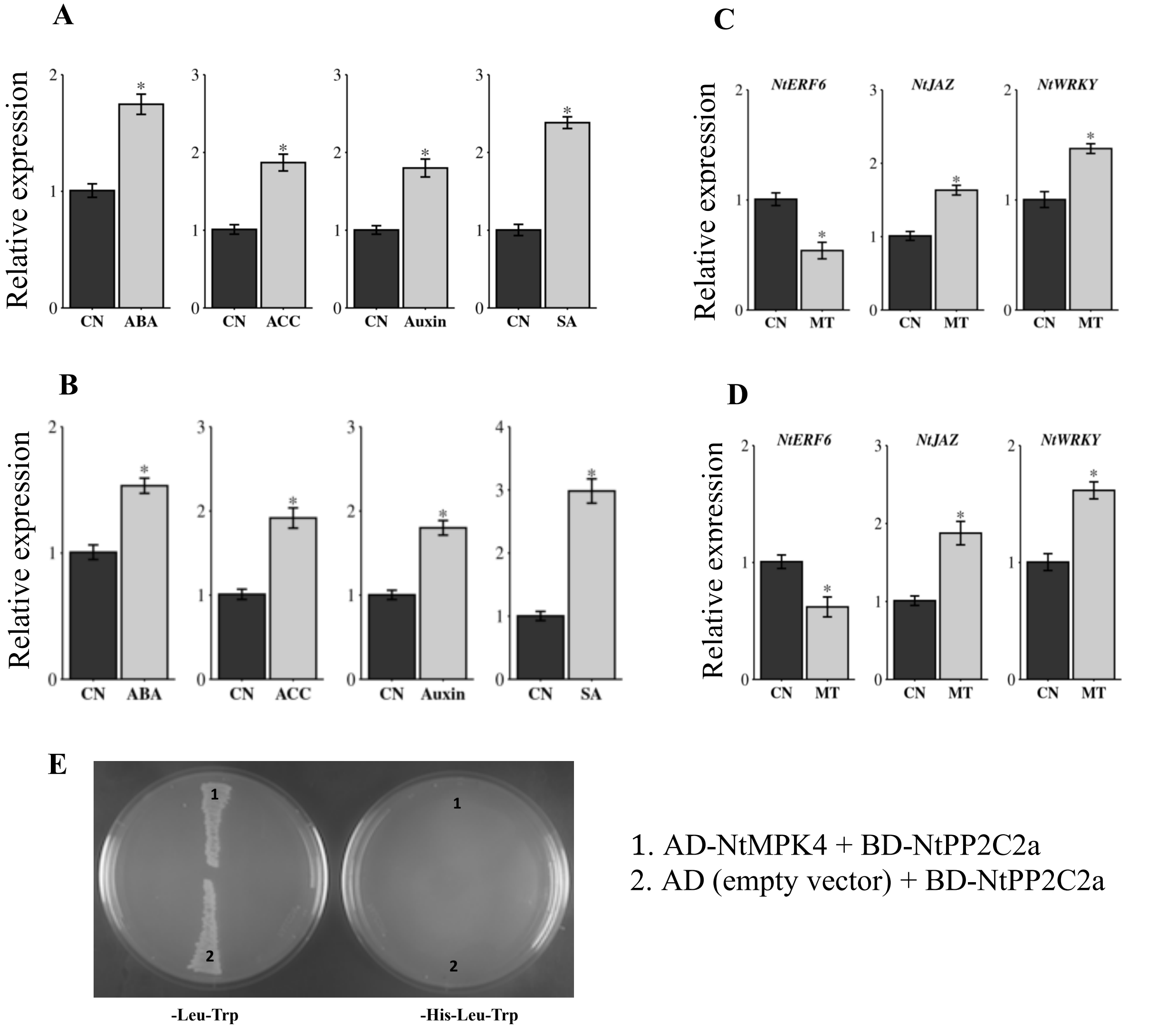

Fig. S3 Relative expression of *NtPP2C2b* in tobacco roots treated with ABA, ACC, auxin and SA. Relative expression of *NtPP2C2b* was measured using RT-qPCR. Tobacco *EF1α* (A) or *α*-tubulin (B) was used as an internal control. Expression of three tobacco transcription factor genes (*ERF6*, *NtJAZ* and *NtWRKY*) in leaves of control (CN) and manually topped (MT) tobacco. Tobacco *EF1α* (C) or *α*-tubulin (D) was used as an internal control. Data represent means±SD of three biological samples. Statistical significance was calculated using the Student's *t-test*: *, P <0.05 ABA, abscisic acid; ACC, 1-aminocyclopropane-1-carboxylic acid; SA, salicylic acid.E. NtPP2C2a does not interact with MPK4 in yeast cells. NtPP2C2b fused to GAL4 DNA binding domain and NtMPK4 fused to GAL4 activation domain were transformed into yeast cell AH109. Protein-protein interaction was tested by the growth of the cells on triple (-his-leu-trp) selection medium.

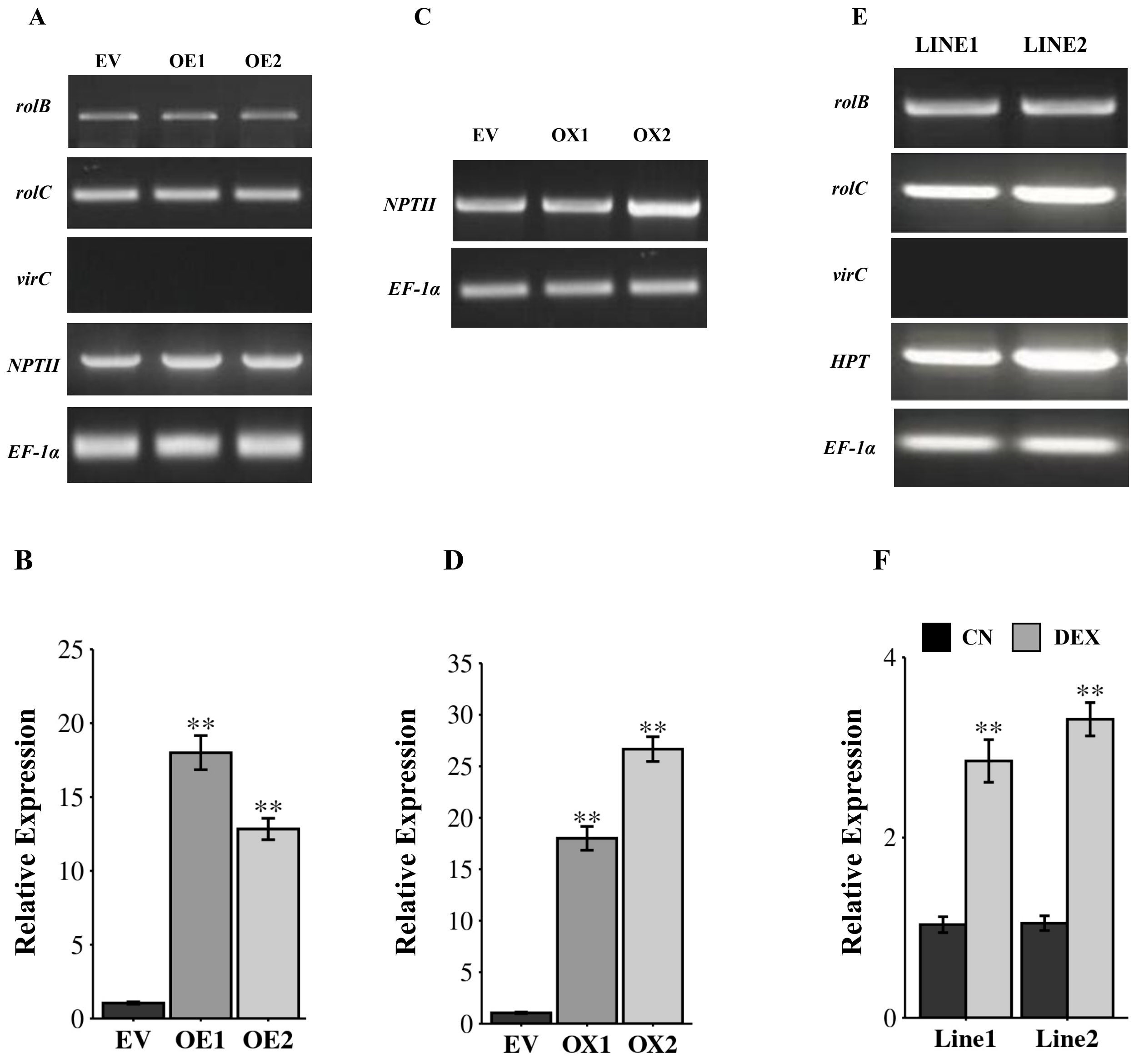

Fig. S4 Molecular analysis of hairy roots and transgenic tobacco plants. Gene-specific primers were used to amplify various fragments in the T-DNA from the empty vector (EV) and *NtPP2C2b*-overexpressing (OE) hairy root lines (OE-1 and OE-2) (**A**), *NtPP2C2b*-overexpressing tobacco plants (EV, OX-1 and OX-2) (**C**), and *NtMPK4*-overexpressing hairy root lines (LINE1 and LINE2) (**E**). Relative expression of *NtPP2C2b* in EV and *NtPP2C2b*-overexpressing hairy roots (OE1 and OE2) (**B**), and transgenic tobacco plants (OX1 and OX2) (**D**). Relative expression of *NtMPK4* in control and DEX-treated *NtMPK4*-overexpressing hairy roots (Line1 and Line2) (**F**).

Relative expression was measured using RT-qPCR. Tobacco *EF1α* was used as an internal control. Statistical significance was calculated using the Student's *t-test*: **, P <0.01. *rolB*, protein-tyrosine phosphatase; *rolC* cytokinin-beta-glucosidase; *virC*, virulence protein; *NPTII*, neomycin phosphotransferase *II*; *EF1*, elongation factor *1α*. CN, control; DEX, dexamethasone.

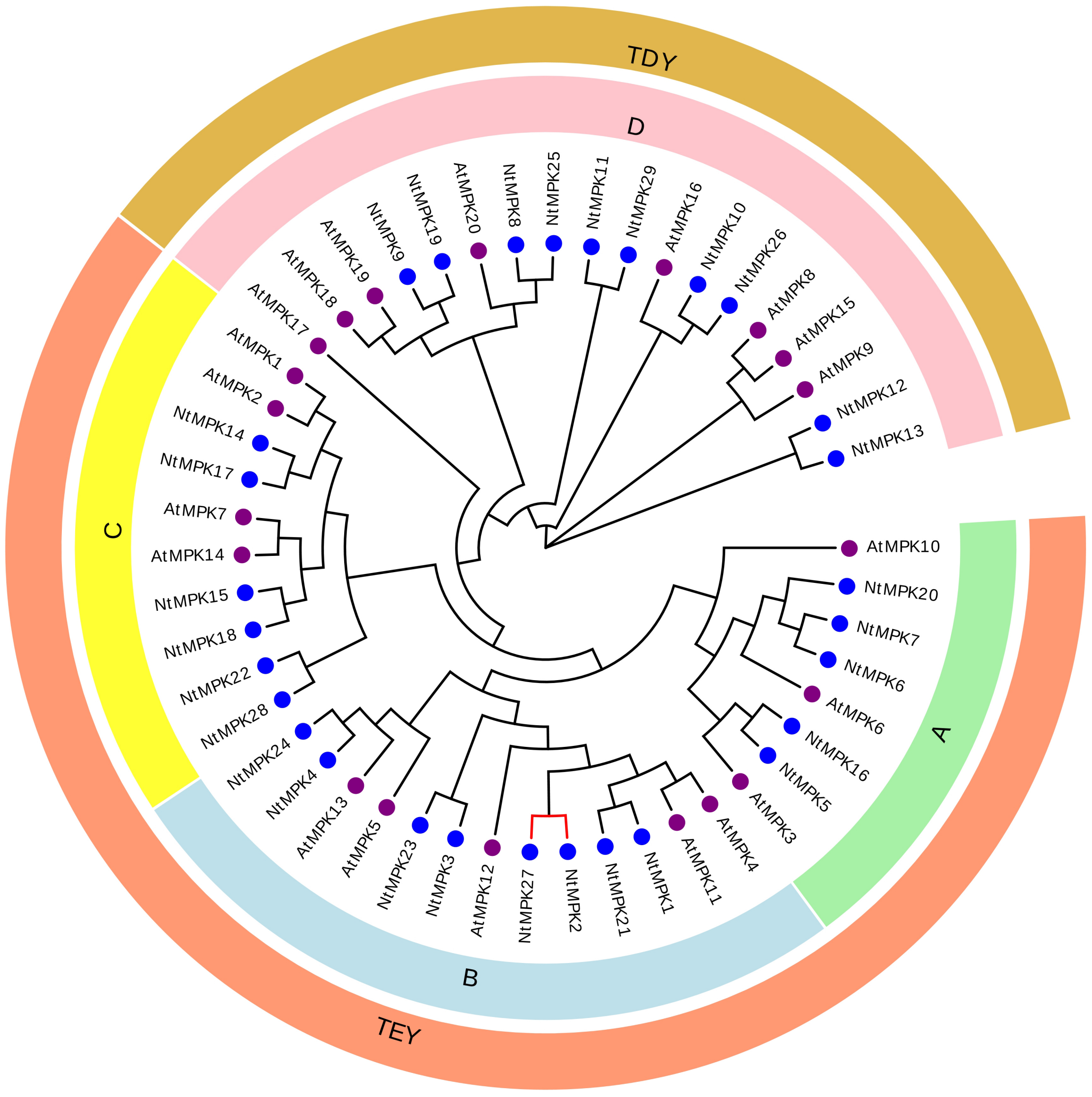

Fig. S5 Phylogenetic analysis of MPKs from tobacco and Arabidopsis. The unrooted phylogenetic tree showing the relationship of MAPK genes from tobacco and Arabidopsis. Tobacco and Arabidopsis MPKs are divided into four groups (A-D). Group A-C contains the TEY motif and group D contains TDY motif in the activation loop. The branch of genes with MEY motif is highlighted with red color. The branch node of tobacco and Arabidopsis genes are indicated by blue and purple, respectively.

A

```
NtMPK4  ..MEAISGDQGVQSNFKGVPTHGGRYAQYNVYGNLFEVSKKYVP.LRPVGRGAYGIVCAAMNSETREEVAIKKIGNAFDNRIDAKRTLREIKLLRHMDHD
AtMPK4  MSAESCFGSSGDQSSSKGVATHGGSYVQYNVYGNLFEVSRKYVPPLRPIGRGAYGIVCAATNSETGEEVAIKKIGNAFDNIIDAKRTLREIKLLKHMDHE

NtMPK4  NVIATKDIIRPPQTENFNDVYIVYELMDTDLHQIIRSNQQLTDDHCRYFLYQILRGLKYIHSANVLHRDLKPSNLFLNANCDLKVGDFGLARTTSETDFM
AtMPK4  NVIAVKDIIKPPQRENFNDVYIVYELMDTDLHQIIRSNQPLTDDHCRFFLYQLLRGLKYVHSANVLHRDLKPSNLLLNANCDLKLGDFGLARTKSETDFM

NtMPK4  TEYVVTRWYRAPELLLNCSEYTAAIDIWSVGCILGEMMTRQPLFPGKDYVHQLKLITELIGSPDDASLGFLRSDNARRYVRQLPQYPRQQFAARFPNSSP
AtMPK4  TEYVVTRWYRAPELLLNCSEYTAAIDIWSVGCILGETMTREPLFPGKDYVHQLRLITELIGSPDDSSLGFLRSDNARRYVRQLPQYPRQNFAARFPNMSA

NtMPK4  GAVDLLEKMLVFDPSRRVTVDQALCHPYLAPLHDINEEPICPKPFSFDFEQPSFTEENIKELIWRESVKFNPDPTH
AtMPK4  GAVDLLEKMLVFDPSRRITVDEALCHPYLAPLHDINEEPVCVRPFNFDFEQPTLTEENIKELIYRETVKFNPQDSV
```

B

```
NtMPK6  MDG.SGQ.QTDTMMSDAGAEQPPTAPQP.VAGMDNIPATLSHGGRFIQYNIFGNIFEVTAKYKPPILPIGKGAYGIVCSALNSETIENVAIKKIANAFDN
AtMPK6  MDGGSGQPAADTEMTEAPGGFPAAAPSPQMPGIENIPATLSHGGRFIQYNIFGNIFEVTAKYKPPIMPIGKGAYGIVCSAMNSETNESVAIKKIANAFDN

NtMPK6  KIDAKRTLREIKLLRHMDHENIVAIRDIIPPPQREAFNDVYIAYELMDTDLHQIIRSNQGLSEEHCQYFLYQILRGLKYIHSANVLHRDLKPSNLLLNAN
AtMPK6  KIDAKRTLREIKLLRHMDHENIVAIRDIIPPPLRNAFNDVYIAYELMDTDLHQIIRSNQALSEEHCQYFLYQILRGLKYIHSANVLHRDLKPSNLLLNAN

NtMPK6  CDLKICDFGLARVTSETDFMTEYVVTRWYRPPELLLNSSDYTAAIDVWSVGCIFMELMDRKPLFPGRDHVHQLRLIMELIGTPSEAEMEFLNENAKRYIR
AtMPK6  CDLKICDFGLARVTSESDFMTEYVVTRWYRAPELLLNSSDYTAAIDVWSVGCIFMELMDRKPLFPGRDHVHQLRLLMELIGTPSEEELEFLNENAKRYIR

NtMPK6  QLPLYRRQSFTEKFPHVHPTAIDLVEKMLTFDPRRRITVEGALAHPYLNSLHDISDEPICMTPFSFDFEQHALTEEQMKELIYRESLAFNPEYQHM
AtMPK6  QLPPYPRQSITDKFPTVHPLAIDLIEKMLTFDPRRRITVLDALAHPYLNSLHDISDEPECTIPFNFDFENHALSEEQMKELIYREALAFNPEYQQ.
```

Fig. S6 Amino acid sequence alignment of Arabidopsis and tobacco MPK4 and MPK6. Alignment of MPK4 (**A**) and MPK6 (**B)** of tobacco (Nt) and Arabidopsis (At) was performed with the CLUSTALO program using the default settings. Identical amino acid residues in the alignment are highlighted in black.

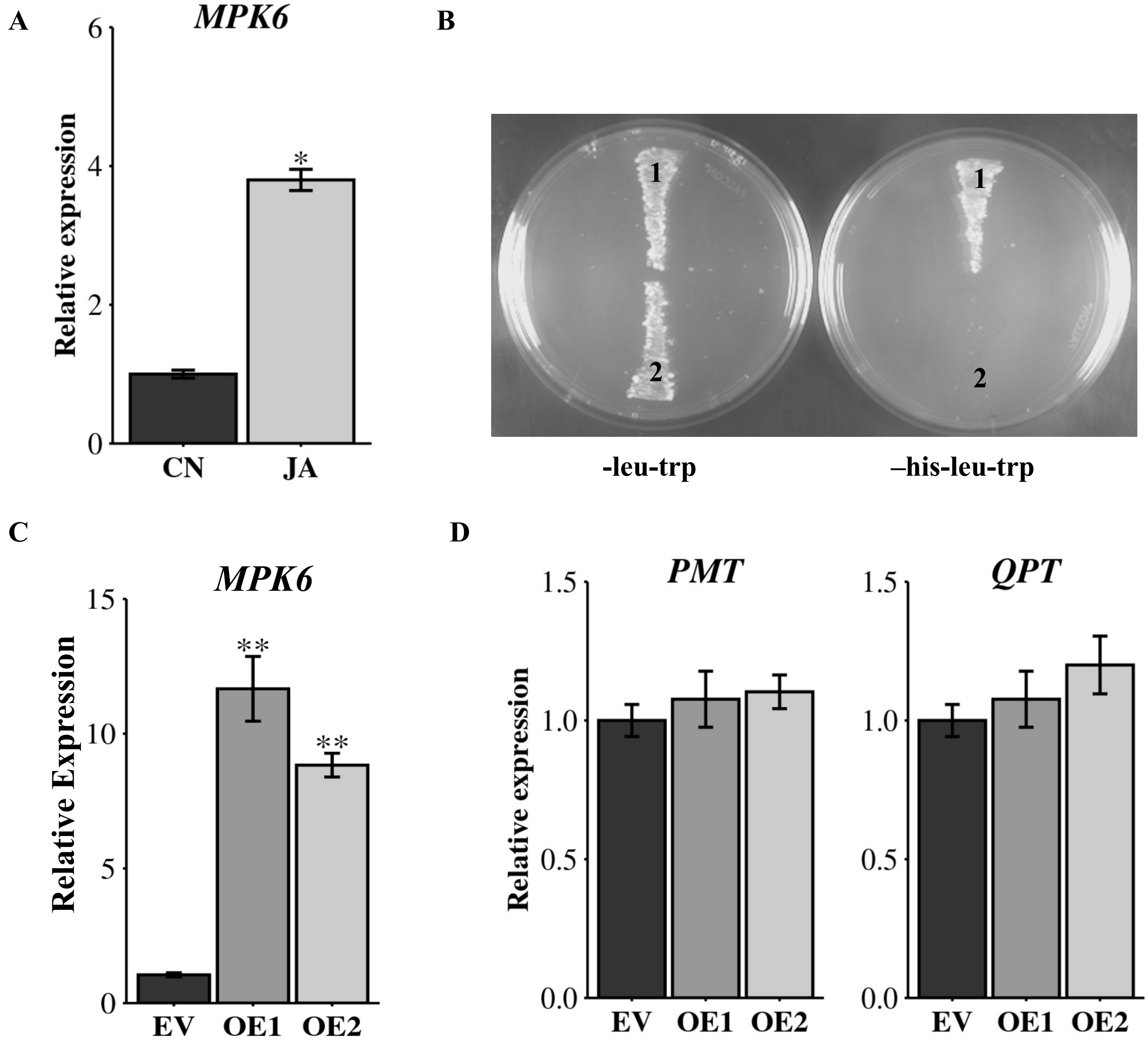

Fig. S7 Expression, protein-protein interaction, and molecular analysis of *NtMPK6* overexpressing hairy roots. **A.** Relative expression of *NtMPK6* in control (CN) and JA-treated roots. Relative expression was measured using RT-qPCR. **B.** NtMPK6 interacts with NtPP2C2b in yeast cells. NtPP2C2b fused to GAL4 DNA binding domain and NtMPK6 fused to GAL4 activation domain were transformed into yeast cell AH109. Protein-protein interaction was detected by the growth of the cells on triple (-his-leu-trp) selection medium. 1. BD-NtPP2C2b + AD-NtMPK6; 2. BD-NtPP2C2b + AD (empty vector). **C.** Relative expression of *NtMPK6* in empty vector (EV) and transgenic hairy roots overexpressing *NtMPK6* (OE1 and OE2). **D.** Relative

expression of *PMT* and *QPT* in EV and transgenic hairy roots overexpressing *NtMPK6* (OE1 and OE2). Relative expression was in measured using RT-qPCR. Tobacco $EF1\alpha$ was used as an internal control. Data represent means±SD of three biological samples. Statistical significance was calculated using the Student's *t-test*: **, $P < 0.01$

Supplement Table 1. Primers used in this study

Gene name	Forward primer (5' - 3')	Reverse primer (5' - 3')
NtPP2C2b	agatctATGCCTTGTGCAATAGCATTAAC	tctagaTTATTGAACAAATTGACTTAGCTGAA
NtMPK4	ggatccATGGAAGCAATTTCAGGTGATC	tctagaTCAGTGAGTTGGATCTGGATTA
NtMPK6	ggatccATGGATGGTTCTGGTCAGCAG	tctagaTCACATATGCTGGTATTCAGG
NtERF221	ggatccATGAATCCCGCTAATGCAACC	ctgcagTTATAGCAACATTTGTAGGTTCC
NtPP2C2b	AATCACCTCTATCGCCGTTG	CCAATCGGAATCGTGATCCT
NtMPK4	TGCCAGTCTTGGATTTCTCC	AGCTCCAGGAGATGAATTGG
NtMPK6	ATCAGGGTTTATCTGAGGAGCA	AGTTGGCATTCAACAGGAGATT
NtMYC2	TGCGATGAAGTCAAGTGGAG	ATTTGCTGGCTTCCTTCCTC
NtERF221	GTTTTGGCAGCTTCCCTTCA	TCGGACTCGGACTTTTCTTC
NtPMT	ATGGCACTTTCGAACATCGG	AGCTCGTTGCCATTGTCATG
NtQPT	TTGATCAATGGGAGGTTTGA	TCAGGGCACCACTAGAAATG
NtBBL	GATGGACGGTTATTAGACCG	ATTTTCCAGGCATAAACAATG
NtA622	GATGGAAATCCCAAAGCAAT	GCAGGTGGTCTCATGTGAAG
NtMATE	TTGCTTGGCAGTGAATGGAC	TGCTGACTTTGAGTGTGCTG
NtJAZ	TTGTAGATTCCGGCAAGGTC	GTTGGTTCAAAACTGCGGTAG
NtEF	ACCACTGGTGGTTTTGAAGC	ACACCAAGGGTGAAAGCAAG
NtTub	GGTATTCAGGTCGGAAATGC	ATCTGGCCATCAGGCTGAAT
rolB	CTTATGACAAACTCATAGATAAAAGGTT	TCGTAACTATCCAACTCACATCAC
rolC	CAACCTGTTTCCTACTTTGTTAAC	AAACAAGTGACACACTCAGCTTC
virC	TTTTGCTCCTTCAAGGGAGGTGCC	GGCTTCGCCAACCAATTTGGAGAT
ntpII	ATGGGGATTGAACAAGATGGA	TCAGAAGAACTCGTCAAGAAG
hpt	ATGAAAAAGCCTGAACTCACCGCGA	CTATTTCTTTGCCCTCGGACGAGTGC
